# Molecular Identification of *Blastocystis* Subtypes in the Cervix: A Study on Polish Patients

**DOI:** 10.3390/jcm14113928

**Published:** 2025-06-03

**Authors:** Barbara Suchońska, Adam Kaczmarek, Maria Wesołowska, Daniel Młocicki, Rusłan Sałamatin

**Affiliations:** 1 1st Department of Obstetrics and Gynaecology, Medical University of Warsaw, 02-015 Warsaw, Poland; 2 Department of Microbiology and Parasitology, Faculty of Medical Science. Collegium Medicum, Cardinal Stefan Wyszyński University in Warsaw, 01-938 Warsaw, Poland; 3 Department of Parasitology and Vector-Borne Diseases, National Institute of Public Health NIH—National Research Institute, 00-791 Warsaw, Poland; 4 Department of Biology and Medical Parasitology, Wrocław Medical University, 50-345 Wrocław, Poland; maria.wesolowska@umw.edu.pl; 5 Department of General Biology and Parasitology, Medical University of Warsaw, 02-004 Warsaw, Poland; daniel.mlocicki@wum.edu.pl

**Keywords:** *Blastocystis*, human, cervix, Poland

## Abstract

**Background**: *Blastocystis* is a common protozoan often found in the gastrointestinal tract of humans. The presence of *Blastocystis* in the female genital tract—has been reported three times, but the genetic material of the protozoan from this site has been identified only once. **Methods**: Patients with cervical erosions were enrolled in the study. Samples were collected using dry swabs. *Blastocystis* DNA was detected using real-time PCR and sequenced. **Results**: Thirty patients with cervical erosions were examined. The presence of *Blastocystis* genetic material was confirmed in nine female patients. *Blastocystis* subtypes ST1, ST6, and ST7 were recovered from the ectocervix and distal part of the cervical canal of women with large, symptomatic glandular ectopies resistant to standard treatment. In one case, we identified a *Blastocystis* subtype ST7 in the material from the cervix, which was different from subtype ST3 found in the anus of the same woman. **Conclusions**: This observation indicates a possible role for *Blastocystis* in cervical erosions and suggests that eradication of these organisms may relieve this condition.

## 1. Introduction

*Blastocystis* are anaerobic protozoans that commonly occur in the human digestive tract [1,2,3,4]. They are characterized by considerable morphological and genetic polymorphisms—with 44 distinct subtypes described to date [5,6,7,8]. In general, the occurrence of *Blastocystis* in humans is estimated to be 10% of the population in developed countries to 100% in developing countries, which is related to the level of hygiene [1,2,3,4,9,10,11].

They are among the group of protozoans that are most commonly detected in fecal samples, but there is considerable controversy regarding their pathogenicity [2,3,12,13,14,15]. There is no consensus on the role of *Blastocystis* in digestive tract diseases [16]. However, some studies suggest that *Blastocystis* may alter the gut microbiome and thus influence processes involved in intestinal inflammation in a protective manner [17]. It is also suggested that outside the digestive tract, the protozoan may become a parasite [18,19].

Currently, it is known that *Blastocystis* does not occur in the vagina or cervix under physiological conditions. Only one report described the presence of *Blastocystis* in vaginal swabs from patients with ‘erosions’ [18].

Recently, two papers have confirmed the presence of *Blastocystis* in the cervix and vagina based on microscopic examination and genetic analysis [19,20]. Villalobos et al. described the presence of *Blastocystis* in the vagina of women and in the semen of men infected with *Trichomonas vaginalis* [20]. This paper sheds new light on the mode of *Blastocystis* transmission. In this paper, we report a rare case of *Blastocystis* detected at an atypical site within the human body—the cervix. To explore the potential source of this unusual infection, we also investigated the presence of *Blastocystis* in the anal region of the affected women.

## 2. Materials and Methods

### 2.1. Study Group and Control Group

The research was performed on patients reporting to the cervical Counselling section of the Outpatient Clinic at the First Department of Obstetrics and Gynecology; they were recruited for the research during routine visits to the clinic. Women who were not menstruating and had not engaged in sexual intercourse within the last 24 h at the time of sample collection were selected.

The study group included 30 regularly menstruating patients of reproductive age (18–50 years old), who had cervical erosion for at least one year, that is, extensive glandular ectopy on the ectocervix, for which attempts at pharmacological treatment had been made. The patients qualified for this group were not pregnant, did not suffer from DM (diabetes mellitus), were not being treated with immunosuppressants, were not taking steroids (which lowers the immune response), and had not used local or systemic metronidazole or cotrimoxazole for the last three months. The control group comprised 30 healthy women of reproductive age without any macroscopic lesions of the cervix, without reported chronic diseases, and without erosion. All patients provided informed consent to participate in the study. A current, normal Pap smear result was also a condition for inclusion in the study. Patient data were anonymized for this study.

The samples were transferred to an employee of the Chair of Biology and Parasitology of the Medical University of Warsaw, and the analysis was performed in the Department of Parasitology and Vector Borne Diseases of the National Institute of Hygiene (Current official name (in English): National Institute of Public Health NIH—National Research Institute), where the material was stored, analyzed, and utilized.

Cervical and anal swabs were collected from patients in the study group. Only cervical swabs were collected from patients in the control group.

### 2.2. Sample Collection

In the study group, samples were collected using two separate dry swabs: one from the ectocervix and the area of the external os of the cervical canal, and the second from the anus at a minimum depth of 1 cm, collecting a minimal amount of fecal material. For the control group, sampling was limited to cervical swabs. We used sterile kits composed of a tube containing a transport medium with added charcoal and a rod with a viscose head and a cork at the opposite end (Deltalab (Rubí Barcelona, Spain), Cat. No. 300285) [21,22]. The test tubes containing the material were transferred to the microbiology laboratory shortly after collection. The samples were stored in a refrigerator at 4 °C until DNA isolation, which was performed on the same or the following day.

### 2.3. Molecular Identification and Sequencing

**Figure 1:**
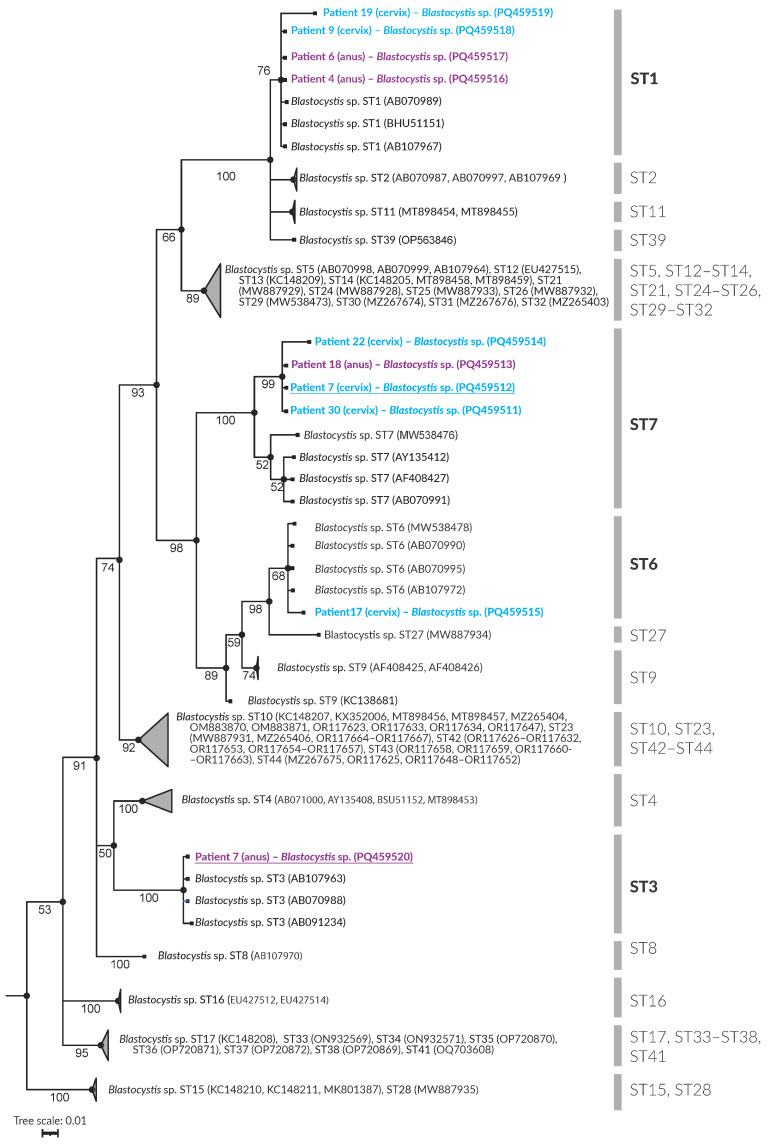
Phylogenetic inference of the 18S rRNA gene partial sequence of *Blastocystis* STs. Bayesian posterior probabilities are shown adjacent to the branch nodes.

DNA was isolated from the samples using Genomic Mini kits (A&A Biotechnology, Gdynia, Poland). A fragment of the small subunit ribosomal RNA (rRNA) was amplified using the Bl18SPPF1 and BL18SR2PP primers and/or the RD5 and BHRDr primers (Table 1, Figure 1).

qPCR amplification was carried out using the Rotor-Gene 6000 system (Corbett Life Science, Paris, France). For each PCR reaction, 2 μL of DNA solution was added, and the total volume of all PCR samples was adjusted to 20 μL. This included 1× concentrated SsoFast™ EvaGreen Supermix and 500 nM of primers. After an initial denaturation step at 95 °C for 5 min, 45 cycles were conducted, with denaturation at 95 °C for 5 s, annealing at 68 °C for 10 s, and extension at 72 °C for 15 s. Signal detection occurred in the green channel at the end of incubation at 72 °C during every cycle.

The resulting products were visualized by electrophoresis on a 2% agarose gel, and the excised bands were purified using a Gel-Out kit (A&A Biotechnology, Gdańsk, Poland). Sequencing was conducted using the Sanger method at the Institute of Biochemistry and Biophysics, Warsaw, Polish Academy of Sciences. Chromatograms were analyzed using CLC Main Workbench v 21.0.6 software (QIAGEN Aarhus A/S, Aarhus, Denmark). The obtained sequences, excluding the primer regions, were compared with those available in the GenBank database using the BLAST tool v. 2.15.0 [26]. Phylogenetic analysis was performed using Bayesian inference [27,28,29], incorporating 104 reference sequences representing *Blastocystis* ST1–ST44 subtypes [7,21,30,31,32] (Table 2). *Proteromonas lacertae* (GenBank: U37108) was used as the outgroup.

The sequences have been deposited in GenBank (accession numbers PQ459511–PQ459520). *Blastocystis* subtype nomenclature is according to Stensvold et al. [33].

This study was approved by the Bioethical Committee of the Medical University of Warsaw (consent number KB1/175/2019; 30 October 2019). All patients provided their written informed consent.

## 3. Results

### 3.1. The Group of Patients with Cervical Erosion

In the group with cervical erosion, *Blastocystis* DNA was detected in both cervical and anal swabs, with varying subtype (STs) distributions.

Phylogenetic analysis (Figure 1) showed that the examined samples corresponded to *Blastocystis* subtypes ST1, ST3, ST6, and ST7. Table 3 provides a detailed overview of *Blastocystis* detection in individual patients. *Blastocystis* was detected in cervical swabs of six patients, while four patients tested positive for the parasite in anal swabs. The specific distribution of the subtypes (STs) is presented in Table 3. *Blastocystis* was present in the swabs of patients 7, 9, 17, 19, 22, and 30. The identified subtypes included ST1, ST6, and ST7.

Anal swabs resulted in four patients [4,6,7,19] testing positive, with identified subtypes ST1, ST3, and ST7. Interestingly, Patient 7 tested positive for *Blastocystis* in both cervical and anal swabs, with different subtypes (ST7 in the cervix and ST3 in the anus).

### 3.2. Control Group

No *Blastocystis* DNA was detected in either the cervical or anal swabs of the control group. This suggests a potential association between cervical erosion and *Blastocystis* colonization or infection in women.

## 4. Discussion

Until recently, only a few authors have confirmed the presence of *Blastocystis* in cervical smears from patients with cervical inflammation. Our molecular findings suggest that *Blastocystis* may colonize the cervical region in certain cases, potentially influenced by erosion, and demonstrate subtype diversity across different anatomical sites.

For a long time, the presence of *Blastocystis* was detected only microscopically [18,34,35,36]. This method was also employed by Escutia-Guzman et al. to confirm the presence of HPV in the cervix [19]. Wołyńska and Soroczan [18] hypothesized that these protozoa could be the etiological factor for non-healing erosions, which have been a therapeutic problem. The presence of this microorganism in 11.5% (47/312) of the analyzed population was accompanied by colpitis in 51% (24/47) and cervical erosion in 32% (15/47). No pathology was found in the genital tract in 17% (8/47) of the cases. Until recently, this was the only paper addressing the parenteral localization of *Blastocystis* and remains the sole study involving such a large group of subjects (319 women), based on which the authors attempted to draw clinical conclusions. PCR analysis allows for an unequivocal diagnosis that is independent of the subjective opinion of the researcher [37,38,39,40,41].

In 2020, a case of extraintestinal *Blastocystis* was published involving a patient with relatively mild symptoms of cervical inflammation [19]. A cytological swab was taken, and the presence of *Blastocystis* sp. was noted microscopically. This was confirmed by PCR analysis of material taken from the anus; however, PCR was not performed on the cervical material. It should not be assumed that the *Blastocystis* present in the anus is identical to that in the genital tract. The presence of *Blastocystis* infection is not routinely assessed in cytological smears, and no appropriate standards have been implemented. The authors suggested that the inability to prove the presence of the protozoan in the cervix was likely due to the topical use of over-the-counter drugs. This may indicate the effectiveness of metronidazole in eradicating the infection, but it could also suggest that the patient’s cervix was not colonized. A clinical case of the parenteral occurrence of *Blastocystis* has been described without any definitive confirmation of this hypothesis. Based on our research, we believe that *Blastocystis* can be present in the cervix, and we have incontestable evidence of this fact. Some of our patients had infections solely in the cervix, while others had infections exclusively in the anus or both locations. It seems that the presence of *Blastocystis* in the anus does not, with any certainty, indicate its presence in the vagina and the cervix. Even if the protozoan is present in both locations, it does not have to be the same subtype. We confirmed the presence of subtypes ST1, ST6, and ST7 in the cervix, whereas subtypes ST1, ST3, and ST7 were present in the anus. In the patient in whom the protozoan was present in both the vagina and the anus, it was determined with absolute certainty that these were two different subtypes: ST7 in the vagina and ST3 in the anus. The confirmation of the presence of *Blastocystis* in the cervix with a simultaneous absence of typical or pronounced clinical symptoms, as observed in our patients, suggests the extraordinary ability of these microorganisms to occupy new niches and adapt to new conditions. Unfortunately, confirming their presence does not equate to identifying the etiological agent for all cervical erosions. It is unclear how to effectively eliminate this infection in patients with such atypical parasite locations.

Bacterial, viral, and fungal infections are common in the vagina and the distal half of the cervix. However, protozoan infections are much less frequent in this area, with *T. vaginalis* being the most common [19,20]. Therefore, colonization of the cervix by *Blastocystis* in a patient with exceptionally mild symptoms (limited to itching) is particularly atypical.

The authors of the most recent paper were able to perform molecular identification of *Blastocystis* subtypes ST1–ST3 in samples taken from the reproductive organs of six women (21.4% of patients tested) and three men (42.8%) infected with *T. vaginalis*, the protozoan responsible for most non-viral sexually transmitted infections. According to the authors, this confirms the great ability of these protozoa to colonize all available niches [19,20].

Similar to the results of our study, Wołyńska, and Soroczan suggested that *Blastocystis* may be present in both the vagina and the anus, but not necessarily simultaneously in the same patient [18]. All women with confirmed infection exhibited symptoms of inflammation or cervical erosion. However, doubts have been raised regarding how *Blastocystis* reaches the cervix and colonizes this area. This may be correlated with an active sex life and/or poor hygiene. The mechanism of autonomous microorganism transfer, or transfer induced by everyday hygiene activities performed by the women themselves through transfer due to the close proximity of the anus and vagina, has been recognized for years in the oral supplementation of gynecological probiotics [42,43,44]. A similar mechanism, though not considered beneficial, could play a role in the transfer of *Blastocystis* and colonization of female genital organs.

Considering that the presence of *Blastocystis* has been confirmed in semen, the possibility of sexual transmission cannot be excluded, especially through vaginal intercourse, with no connection to neglecting hygiene.

In summary, *Blastocystis* can occur not only in the alimentary tract and anus but also in other locations in the anogenital area. In our study, *Blastocystis* was found in the cervixes of six out of thirty (20%) patients who had extensive non-healing erosions and in none of the control groups who had no erosions. Based on the literature data, we expected to find *Blastocystis* in cervixes affected by erosions. Therefore, using genetic analysis and sequencing, we confirmed the results of Wołyńska and Soroczan.

Similar to other researchers studying *Blastocystis*, we could not conclusively determine whether these protozoans are commensals or parasites at this cervical location. To date, research has not been conducted on a sufficiently large patient group to assess the extent of this phenomenon and the true frequency of *Blastocystis* co-occurrence with cervical lesions. In our pilot study, which involved a relatively small sample of patients, we sought to determine whether *Blastocystis* colonization of the cervix could be confirmed using molecular methods and whether it occurred more frequently in women with erosions. We found positive answers to both questions. However, the issue of whether treatment is necessary, and if so, how it should be approached, remains unresolved and requires further investigation.

## Figures and Tables

**Table 1:** List of primers and sizes of amplified products.

Primers	Sequences	Size of Product	Sources
RD5	5′-GGA AGC TTA TCT GGT TGA TCC TGC CAG TA-3′	ca. 560 bp	[23]
BHRDr	5′-GAG CTT TTT AAC TGC AAC AAC G-3′		[24]
Bl18SPPF1	5′-AGT AGT CAT ACG CTC GTC TCA AA-3′	ca. 340 bp	[25]
BL18SR2PP	5′-TCT TCG TTA CCC GTT ACT GC-3′		

bp—base pair; ca.—circa (approximately).

**Table 2:** GenBank reference sequences used in the construction of phylogenetic tree.

Subtype	Number in the GenBank Database
ST1	AB070989 (human), U51151 (human), AB107967 (monkey)
ST2	AB070987 (human), AB070997 (monkey), AB107969 (monkey)
ST3	AB070988 (human), AB091234 (human), AB107963 (pig)
ST4	AB071000 (rat), AY135408 (rat), MT898453 (human), U51152 (guinea pig)
ST5	AB070999 (pig), AB107964 (pig), AB070998 (pig)
ST6	AB107972 (partridge), AB070995 (quail), AB070990 (human), MW538478 (chicken)
ST7	AY135412 (duck), AB070991 (human), AF408427 (human), MW538476 (chicken)
ST8	AB107970 (black-and-white ruffed lemur)
ST9	AF408425 (human), AF408426 (human), KC138681 (human)
ST10	KX352006 (water), KC148207 (dromedary), MT898456 (cattle), MT898457 (cattle), MZ265404 (goat), OM883870 (muskox), OM883871 (sheep), OR117623 (cattle), OR117633 (goat), OR117634 (goat), OR117647 (sheep)
ST11	MT898454 (elephant), MT898455 (elephant)
ST12	EU427515 (northern swamp wallaby)
ST13	KC148209 (java mouse-deer)
ST14	KC148205 (cattle), MT898458 (cattle), MT898459 (cattle)
ST15	KC148210 (dromedary), KC148211 (gibbon), MK801387 (pig)
ST16	EU427512 (red kangaroo), EU427514 (red kangaroo)
ST17	KC148208 (common gundi)
ST21	MW887929 (white-tailed deer)
ST23	MW887931 (cattle), MZ265406 (goat), OR117664–OR117667 (goat)
ST24	MW887928 (white-tailed deer)
ST25	MW887933 (cattle)
ST26	MW887932 (cattle)
ST27	MW887934 (indian peafowl)
ST28	MW887935 (indian peafowl)
ST29	MW538473(chicken)
ST30	MZ267674 (white-tailed deer)
ST31	MZ267676 (white-tailed deer)
ST32	MZ265403 (goat)
ST33	ON932569 (horse)
ST34	ON932571 (horse)
ST35	OP720870 (human)
ST36	OP720871 (bat)
ST37	OP720872 (heteromyd)
ST38	OP720869 (water vole)
ST39	OP563846 (monkey)
ST41	OQ703608 (human)
ST42	OR117626–OR117632 (cattle), OR117653 (red-deer), OR117654–OR117657 (cattle)
ST43	OR117658 (goat), OR117659 (goat), OR117660–OR117663 (sheep)
ST44	MZ267675 (white-tailed-deer), MZ267679 (white-tailed-deer), OM883872 (sheep), OR117624 (cattle), OR117625 (cattle), OR117648–OR117652 (sheep)

**Table 3:** *Blastocystis* presence in swabs taken from the cervix and anus.

Patients	Group of Women with Erosion	
	Cervix	Anus
Patient 4	–	+ (ST1)
Patient 6	–	+ (ST1)
Patient 7	+ (ST7)	+ (ST3)
Patient 9	+ (ST1)	–
Patient 17	+ (ST6)	–
Patient 18	–	+ (ST7)
Patient 19	+ (ST1)	–
Patient 22	+ (ST7)	–
Patient 30	+ (ST7)	–

‘+’ indicates detection of *Blastocystis*; ‘–’ indicates no detection. The subtype of *Blastocystis* identified is shown in parentheses.

## Data Availability

The dataset from this study is held securely in coded form at the Medical University of Warsaw. Data-sharing agreements prohibit making the dataset publicly available. However, the data can be made available upon reasonable request to the corresponding author (RS) after obtaining the necessary ethical and data sharing approvals.
